# Ergosterol as a natural modulator of intestinal cholesterol absorption via NPC1L1: an *in silico* insight into hypercholesterolemia

**DOI:** 10.3389/fbinf.2026.1734995

**Published:** 2026-04-10

**Authors:** Aparna G. Shenoy, Amrithesh Sureshkumar, Vishal Ravi, Rajesh Raju, Niyas Rehman

**Affiliations:** Centre for Integrative Omics Data Science (CIODS), Yenepoya (Deemed to be University), Mangalore, Karnataka, India

**Keywords:** cardiovascular disease, cholesterol uptake inhibition, drug discovery, ergosterol, *in-silico* analysis, NPC1L1

## Abstract

**Introduction:**

Cardiovascular disease (CVD) is the leading cause of death worldwide and is substantially caused by high cholesterol levels. High levels of low-density lipoprotein cholesterol (LDL-C) lead to atherosclerosis, which greatly raises the risk of heart disease, such as heart attack, stroke, and so on. Therapeutic strategies for lowering cholesterol levels include the use of statins and PCSK9 inhibitors. To further reduce intestinal cholesterol absorption, the combination of ezetimibe with statin is an effective approach for controlling LDL-C levels and reducing cardiovascular disease risk. The Niemann-Pick C1-Like 1 (NPC1L1) protein is an important regulator of cholesterol absorption in the intestines and is a clinically proven target for lowering LDL-C levels. Although ezetimibe is prescribed for hypercholesterolemia, which blocks NPC1L1-mediated cholesterol absorption, its prolonged use could lead to hepatotoxicity, prompting the search for safer alternatives.

**Objective:**

To explore the interaction of ergosterol with NPC1L1 and assess its potential to modulate cholesterol absorption using *in silico* methods.

**Methods:**

Molecular docking, ADMET profiling, molecular dynamics (MD) simulations, post-MD analysis, and MM-PBSA binding free energy calculations were conducted to study ergosterol–NPC1L1 interactions and compare them with NPC1L1-ezetimibe and NPC1L1-cholesterol complex.

**Results:**

Docking and ADMET analyses revealed the existence of robust hydrophobic interaction of ergosterol with the binding pocket of NPC1L1 (binding energy −11.941 kcal/mol), identical to the binding of ezetimibe (−12.38 kcal/mol) and cholesterol (−11.432 kcal/mol), exhibiting comparable binding energies along with favourable pharmacokinetic properties. Molecular dynamics (MD) simulations and post-MD trajectory analyses suggest that the NPC1L1-ergosterol complex maintains structural stability during the simulation period. MM-PBSA binding free energy calculations consistently suggested a strong and stable interaction between NPC1L1-ergosterol (−27.61 ± 3.72 kcal/mol), comparable to that of NPC1L1-ezetimibe (−19.42 ± 4.87 kcal/mol) and NPC1L1-cholesterol (−32.53 ± 3.71 kcal/mol) complex.

**Conclusion:**

Our computational modelling studies suggested that ergosterol forms energetically favourable interactions with NPC1L1, hinting at its potential as a candidate for hypercholesterolemia and CVD management. However, comprehensive preclinical and clinical investigations are required to substantiate its therapeutic relevance.

## Graphical Abstract

**Figure:**
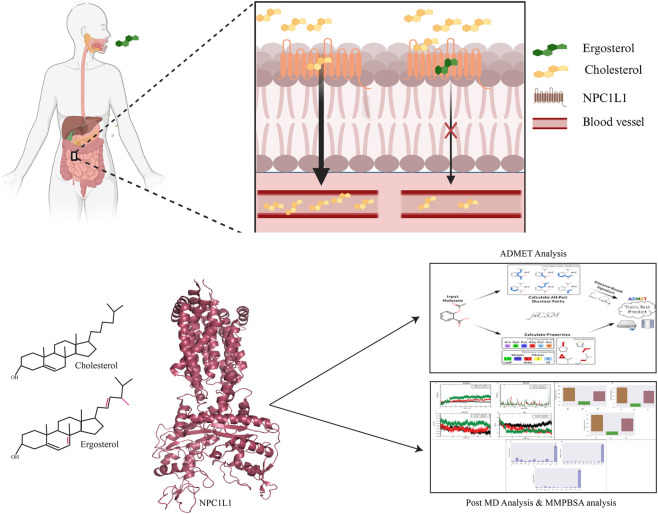
Created in https://BioRender.com.

## 1 Introduction

Cardiovascular diseases (CVDs) are one of the leading global health concerns, contributing to approximately 17.9 million deaths annually, which accounts for nearly one-third of worldwide mortality (World Health Organization (WHO), 2024). Hypercholesterolemia, characterised by elevated plasma cholesterol and LDL-C levels with reduced HDL-C levels, is a major contributor to CVD (Duan et al., 2022; From the American Association of Neurological Surgeons ASoNC et al., 2018; Huang et al., 2020; Jia et al., 2011). Cholesterol is an important part of the cell membrane. It helps maintain membrane fluidity, stability, and permeability, and plays a key role in cell organization and signaling. It also serves as a precursor for vitamin D, bile acids, and steroid hormones (Zhang et al., 2022a; Howles, 2016). However, dysregulation of cholesterol metabolism, whether through excessive dietary absorption, disrupted biosynthesis, or impaired bile acid metabolism, promotes lipid accumulation, inflammation, and plaque deposition within artery walls, which leads to the development of CVD (Martin et al., 2024).

To address elevated cholesterol and its associated risks, treatment strategies focus on reducing plasma cholesterol levels by reducing endogenous cholesterol synthesis, enhancing elimination, or inhibiting intestinal cholesterol absorption. While statins constitute first-line therapy, additional options such as ezetimibe, PCSK9 inhibitors, bile acid sequestrants, and bempedoic acid are often employed alongside lifestyle measures to enhance efficacy. Statins such as pitavastatin, rosuvastatin, lovastatin, pravastatin, and fluvastatin (Tsujita et al., 2023; Shi and Han, 2025) exert their lipid-lowering effects by inhibiting HMG-CoA reductase; however, they do not directly target intestinal cholesterol absorption (Hashemi et al., 2017; Tobert, 2003; Istvan and Deisenhofer, 2001).

This limitation has redirected therapeutic strategies towards targeting intestinal cholesterol absorption, identifying niemann-pick c1-like 1 (NPC1L1) as a clinically proven target whose inhibition drastically reduces intestinal cholesterol uptake and cardiovascular risk, underscoring its therapeutic value in hypercholesterolemia (Betters and Yu, 2010; Valdivia et al., 2024).

NPC1L1 protein is a transmembrane cholesterol transporter localized at the brush border membrane of small intestinal enterocytes, where it mediates uptake of dietary and biliary cholesterol (Jia et al., 2011; Park, 2013; Temel et al., 2007; Altmann et al., 2004). Upregulation of NPC1L1 markedly enhances intestinal cholesterol absorption, exacerbating hypercholesterolemia and cardiovascular risk; thus, targeting NPC1L1 downregulation may offer a viable therapeutic strategy (Luo et al., 2020; Jia et al., 2021). Currently, ezetimibe is the only FDA-approved NPC1L1 inhibitor and has proven efficacy in reducing plasma cholesterol and cardiovascular events, particularly when combined with statins (Gagne et al., 2002; Sudhop et al., 2002; Davis et al., 2001; Zhang et al., 2022b). However, its use is associated with potential adverse effects, including severe hepatic toxicity, myalgia, myopathy, arrhythmias, gallstone formation, and various gastrointestinal disturbances. The need for long-term therapy further amplifies these safety concerns (Kante et al., 2025; Oza et al., 2025; Stolk, 2006).

Ergosterol, the predominant sterol in fungal cell membranes and a dietary component of edible mushrooms, has been reported to exhibit antioxidant, anti-inflammatory, and cholesterol-lowering properties (Das and Gurusiddaiah, 2023; Cardwell et al., 2018; He et al., 2019; Ku et al., 2011). Structurally, ergosterol closely resembles cholesterol, differing only in side-chain composition and degree of unsaturation (Hung et al., 2016; Hsueh et al., 2007). This structural resemblance may enable ergosterol to interfere with intestinal cholesterol absorption, thereby promoting faecal sterol excretion and reducing plasma cholesterol levels, warranting further investigation into its underlying mechanism of action (He et al., 2019; Yang et al., 2024; El Omari et al., 2024).

In this study, we investigated the potential interaction of ergosterol with NPC1L1, based on the hypothesis that cholesterol analogues might competitively bind to NPC1L1 and potentially inhibit cholesterol transport. Molecular docking was conducted to determine the binding affinity and identify the binding site of the ergosterol-NPC1L1 complex, followed by molecular dynamics (MD) simulations and subsequent analysis to evaluate its stability and conformational behaviour. This integrated computational method aims to elucidate the molecular basis of the NPC1L1-ergosterol interaction and to investigate its potential as a safer, biologically active treatment alternative for managing hypercholesterolemia and related cardiovascular conditions.

## 2 Materials and methods

### 2.1 Protein preparation

The cryo-electron microscopy structure of human niemann-pick c1-like 1 (NPC1L1) in complex with ezetimibe (PDB ID: 7DFZ, 3.58 Å resolution) was retrieved from RCSB PDB (Hu et al., 2021). Even though there are multiple crystal and cryo-EM structures of NPC1L1 available, only 7DFZ contains NPC1L1 in complex with ezetimibe. Therefore, 7DFZ structure was selected for the present study to ensure accurate representation of the experimentally validated NPC1L1–ezetimibe binding complex. Structural refinement was conducted using Schrödinger’s Maestro 2024.3 protein preparation wizard (Sastr et al., 2013) with default parameters, encompassing the removal of solvent molecules, addition of hydrogen atoms, removal of heteroatoms, and addition of missing residues using the prime module. Energy minimization was performed using the OPLS4 force field (Sindhu et al., 2024).

### 2.2 Molecular docking

Ergosterol and cholesterol were selected as the ligands. The three-dimensional structure (.sdf format) of ergosterol (Drug Bank ID: DB04038) was retrieved from the Drug Bank database (Drug Bank, 2025). The cholesterol molecule co-crystallized with the NPC1L1 protein was extracted directly from the same protein crystal structure for comparative analysis. The receptor grid was generated by centering it at the binding site of the control drug ezetimibe. The grid centre coordinates were set to X = 173.33, Y = 176.32, Z = 184.22, with a length of 20 Å to encompass the active site. The ligands were docked to the target protein using the extra precision (XP) docking mode to ensure high accuracy in pose prediction. The OPLS4 force field was applied for energy minimization and optimization of docking poses, ensuring reliable interaction energies and geometries (Lu et al., 2021). 2D interaction of the protein-ligand complex was visualized using the Ligand interaction tool in Schrodinger Maestro.

### 2.3 Absorption, distribution, metabolism, excretion, and toxicity (ADMET) analysis

Ezetimibe and ergosterol were evaluated for their ADMET properties (absorption, distribution, metabolism, excretion, and toxicity) using the pk-CSM web tool (https://biosig.lab.uq.edu.au/pkcsm/). SMILES for each compound were submitted as input for the computer prediction of pharmacokinetic and toxicological characteristics. Gut localised actions of both the compounds were analysed, which include parameters such as human intestinal absorption, total clearance, and hepatotoxicity. Along with that, other parameters such as steady-state volume of distribution (VDss), substrate potential, and inhibitory activity across various cytochrome P450 (CYP) isoforms, maximum tolerated dose in humans, oral rat acute toxicity (LD50), and oral rat chronic toxicity (LOAEL) were analysed. These parameters were selected for initial screening and comparative pharmacokinetic evaluation of the compounds.

### 2.4 Molecular dynamics (MD) simulation

To investigate the conformational dynamics within the drug-binding pocket, molecular dynamics (MD) simulations of NPC1L1-ezetimibe, NPC1L1-cholesterol, and NPC1L1-ergosterol were carried out for 200 ns using GROMACS 2023.4 (Abraham et al., 2015). The system was parameterized, and protein topology was generated using the CHARMM36 (July 2022) all-atom force field (Yu et al., 2021), which provides accurate descriptions for proteins, ligands, and ions. The protein was modelled using standard CHARMM36 parameters, and ligand parameters were generated using the CHARMM General Force Field (CGenFF), ensuring compatibility within the CHARMM framework. The ligand parameters were obtained from the Swiss Param server (Abraham et al., 2015). The system was embedded in a triclinic simulation box with a minimum spacing of 1.0 nm between the protein surface and the box boundary and solvated with TIP3P water molecules. System neutralization and ionic strength adjustment were achieved by adding Na^+^ ions. Energy minimization was first carried out using the steepest descent algorithm until convergence to remove steric clashes. Equilibration was conducted in two stages, which included NVT (constant Number, Volume, Temperature) equilibration, where the system was equilibrated at 300 K under constant volume conditions for 100 ps Temperature coupling was achieved using the V-rescale (modified Berendsen) thermostat with a coupling time constant of 0.1 ps, ensuring stable and accurate temperature control. NPT (constant Number, Pressure, Temperature) equilibration was followed by 100 ps of constant pressure conditions at 1 bar. Pressure coupling was performed using the Parrinello–Rahman barostat with a coupling time constant of 2.0 ps, allowing isotropic pressure scaling and correct density relaxation.

All covalent bonds involving hydrogen atoms were constrained using the LINCS algorithm, enabling the use of a 2-fs time step. Long-range electrostatic interactions were treated using the Particle Mesh Ewald (PME) method, with a real-space cutoff of 1.2 nm, and the same cutoff was applied to van der Waals interactions.

### 2.5 Post MD analysis

Post-MD simulation trajectory analyses were conducted using GROMACS’s integrated analysis tools. This includes root mean square deviation (RMSD) to assess overall structural stability relative to the reference conformation, root mean square fluctuation (RMSF) to characterize residue-level flexibility, radius of gyration (Rg) to monitor the compactness and potential conformational changes of the complexes, and solvent accessible surface area (SASA) to quantify changes in solvent exposure of the molecular surface throughout the simulation.

### 2.6 Molecular mechanics poisson-boltzmann surface area (MM-PBSA) calculation

MM-PBSA was used to estimate the binding free energy (ΔG-bind) and per-residue energy decomposition profiles of NPC1L1-ezetimibe, NPC1L1-cholesterol, and NPC1L1-ergosterol complexes for 200 ns. The g_mmpbsa tool was employed, and a total of 1000 evenly spaced simulation frames were extracted for binding energy estimation (Mangat et al., 2022). Per-residue energy decomposition was also performed to identify key residues contributing to ligand binding. The MMPBSA_ana module was utilized for analysis and visual representation of the calculated energies.

## 3 Results

### 3.1 Molecular docking and protein-ligand interaction

Protein–ligand docking was performed to determine the binding sites of NPC1L1 with ezetimibe, cholesterol, and ergosterol. Ligand orientations within the binding pocket were identified by visualizing the 3D docked poses. Two-dimensional interaction diagrams revealed that a substantial number of hydrophobic contacts were formed between binding site residues and ligand moieties.

The NPC1L-ezetimibe complex exhibited an XP docking score of −12.38 kcal/mol. The 2D interaction revealed hydrogen bond with LEU875, along with extensive hydrophobic interactions involving VAL380, LEU382, TRP383, ILE525, LEU621, VAL667, ILE698, VAL701, ALA768, VAL769, PHE772, LEU871, LEU875, ALA876, PHE1101, TYR1102, VAL1160, LEU1234, PHE1238, and PHE1239. Additionally, GLN873 forms a polar interaction with NPC1L1.

The NPC1L1-cholesterol complex exhibited an XP docking score of −11.432 kcal/mol, indicating a strong binding affinity. The interaction analysis revealed that TYR1102 formed a hydrogen bond with cholesterol, while multiple residues, including VAL380, LEU382, TRP383, LEU621, ILE625, VAL697, ILE698, VAL701, ALA768, VAL769, PHE772, LEU871, ALA876, PHE1101, VAL1166, ILE1169, LEU1234, and PHE1239, participated in hydrophobic interactions, contributing to the stability of the complex. Additionally, GLN873 formed a polar interaction, further stabilizing the ligand–protein binding interface.

**FIGURE 1:**
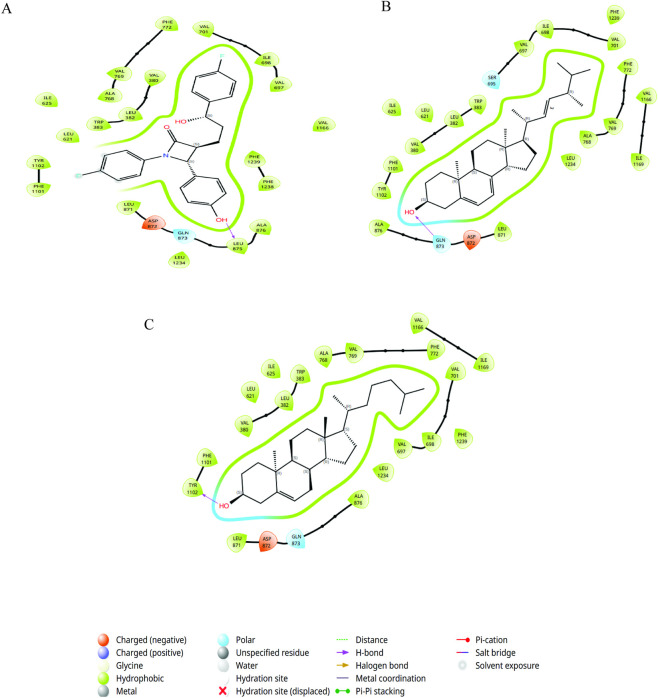
2-Dimensional interaction of **(A)** NPC1L1-ezetimibe and **(B)** NPC1L1-cholesterol and **(C)** NPC1L1-ergosterol complex.

The NPC1L1-ergosterol complex exhibited an XP docking score of −11.941 (kcal/mol). Interaction analysis identified a polar interaction with SER695 and a hydrogen bond with GLN873. In addition, multiple hydrophobic contacts were identified with VAL380, LEU382, TRP383, LEU621, ILE625, VAL697, ILE698, VAL701, ALA768, VAL769, PHE772, LEU871, ALA876, PHE1101, TYR1102, VAL1166, ILE1169, LEU1234, and PHE1239. Interactions of the NPC1L1 complex, along with the docking score, are presented in Table 1 and illustrated in Figure 1.

**TABLE 1:** Docking score and residue interaction of the NPC1L1-ezetimibe, NPC1L1-cholesterol and NPC1L1-ergosterol complexes.

Compound	Docking score (XP) (kcal/mol)	Hydrogen bond	Other interactions
NPC1L1-ezetimibe	−12.38	LEU875	VAL380, LEU382, TRP383, ILE525, LEU621, VAL667, ILE698, VAL701, ALA768, VAL769, PHE772, LEU871, LEU875, ALA876, PHE1101, TYR1102, VAL1160, LEU1234, PHE1238, and PHE1239 (hydrophobic bond); GLN873 (polar interaction)
NPC1L1-cholesterol	−11.432	TYR1102	VAL380, LEU382, TRP383, LEU621, ILE625, ILE698, VAL701, ALA768, VAL769, PHE772, LEU871, ALA876, PHE1101, VAL1166, ILE1169, LEU1234, PHE1239 (hydrophobic bond); GLN873 (polar interaction)
NPC1L1-ergosterol	−11.941	GLN873	VAL380, LEU382, TRP383, LEU621, ILE625, VAL697, ILE698, VAL701, ALA768, VAL769, PHE772, LEU871, ALA876, PHE1101, TYR1102, VAL1166, ILE1169, LEU1234, and PHE1239 (hydrophobic bonds); SER695 (polar interaction)

### 3.2 Absorption, distribution, metabolism, excretion, and toxicity (ADME/T) analysis

The *in silico* ADMET analysis of ergosterol and cholesterol in comparison with ezetimibe indicates a generally favourable pharmacokinetic and safety profile, supporting its potential as a therapeutic drug. Evaluation against Lipinski’s Rule of Five shows ergosterol to be more compliant, supporting better drug-likeness. Human intestinal absorption for ergosterol was 95.41%, which is higher than that of ezetimibe, indicating improved oral bioavailability. Total clearance values indicate that ergosterol may be rapidly eliminated compared to ezetimibe and no hepatotoxicity was predicted. The steady-state volume of distribution (VDss) was similarly higher for ergosterol (1.088 log L/kg), which means it was spread out throughout a wider range of tissues. Ergosterol and ezetimibe were both predicted not to be substrates of CYP2D6. In addition, ergosterol was predicted not to inhibit CYP1A2, CYP2C19, CYP2C9, CYP2D6, or CYP3A4, while ezetimibe was assumed to inhibit CYP2C9, CYP2C19, and CYP3A4. The maximum tolerated dose (MTD) for ergosterol suggests slightly lower tolerability, and oral rat acute toxicity (LD50) values indicate that ezetimibe (2.464 log mol/kg) is less acutely toxic than ergosterol (2.034 log mol/kg), reflecting a higher tolerated single-dose exposure. However, administration of ergosterol at lower doses may alleviate potential toxicity concerns. Overall, ergosterol exhibits favourable pharmacokinetic properties. ADMET analysis is provided in Table 2 (refer to Supplementary Table S1 for detailed ADMET analysis).

**TABLE 2:** Comparative ADMET analysis of ezetimibe, cholesterol, and ergosterol, focusing on their pharmacokinetic and toxicological characteristics.

Properties	Ezetimibe	Ergosterol	Cholesterol
Molecular weight	409.432	396.659	386.664
LogP	4.8883	7.3308	7.3887
Intestinal absorption (human)	91.013	95.41	93.937
VDss (human)	−0.436	0.272	0.244
CYP2D6 substrate	No	No	No
CYP3A4 substrate	Yes	Yes	Yes
CYP1A2 inhibitor	No	No	No
CYP2C19 inhibitor	Yes	No	No
CYP2C9 inhibitor	Yes	No	No
CYP2D6 inhibitor	No	No	No
CYP3A4 inhibitor	Yes	No	No
Total clearance	−0.334	0.564	0.589
Max. Tolerated dose (human)	−0.092	−0.691	−0.74
Oral rat acute toxicity (LD50)	2.172	2.255	2.299
Hepatotoxicity	Yes	No	No

Reference: Human intestinal absorption below 30% signifies inadequate uptake, but readings beyond this threshold indicate efficient absorption. Volume of dispersion (VD) below −0.15 signifies low dispersion, while values beyond 0.45 reflect high dispersion. LogBB values more than 0.3 indicate effective blood-brain barrier (BBB) penetration, but values less than −1.0 imply inadequate brain distribution. CNS penetration is anticipated when values surpass −2.0; nevertheless, substances with values below −3.0 are deemed incapable of traversing the blood-brain barrier. The maximum tolerable dose is categorised as low at ≤0.477 log(mg/kg/day) and high for values exceeding this threshold.

### 3.3 Molecular dynamics (MD) simulation

A molecular dynamics simulation is used to assess the stability and flexibility of the system throughout a simulation of 200 ns. To confirm proper minimization and equilibration before MD run, we have analysed total energy profiles, temperature profiles, and pressure profiles during NVT and NPT equilibration (detailed information is provided in Supplementary Material S1 under the heading ‘Validation of Minimization and Equilibration of MD Simulation’, along with Supplementary Figure S1).

### 3.4 Post MD analysis

Molecular dynamics (MD) simulations were performed for NPC1L1-ezetimibe, NPC1L1-ergosterol and NPC1L1-cholesterol complexes to analyse their structural dynamics. Key parameters, including stability, flexibility, compactness, and solvent exposure, were examined to compare ergosterol’s binding characteristics against those of cholesterol and the established inhibitor ezetimibe. For further analysis, the running average over 100 frames for RMSD, RMSF, Rg, and SASA was calculated and is provided in Supplementary Material S1 under the heading ‘Running Average (100 Frames) Analysis’, along with Supplementary Figure S2.

**FIGURE 2:**
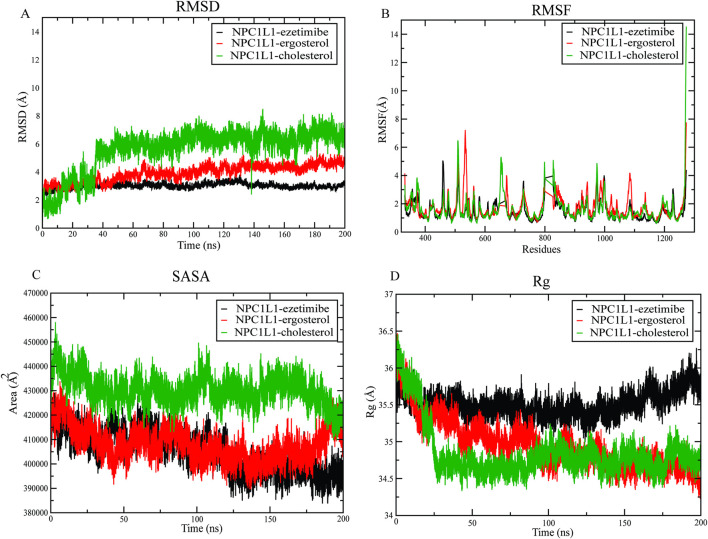
Structural stability and dynamic behaviour of the NPC1L1-ezetimibe, NPC1L1-ergosterol, and NPC1L1-cholesterol complexes over a 200 ns MD simulation. A plot of **(A)** root mean square deviation (RMSD), **(B)** root mean square fluctuation (RMSF), **(C)** solvent accessible surface area (SASA), and **(D)** radius of gyration (Rg), plots.

The NPC1L1-ezetimibe complex (black) demonstrated a stable RMSD trajectory, with an average deviation of around 3.0 Å with minimal fluctuations throughout the simulation (Figure 2A). The NPC1L1-cholesterol complex (green) displayed a behaviour similar to that of ergosterol. RMSD showed initial fluctuations, with values ranging from 1.5 Å to 6.2 Å between 20 and 40 ns before stabilisation. Similarly, NPC1L1-ergosterol complex (red) exhibited a comparable RMSD range between 1.0 and 4.2 Å, indicating consistent stability during the simulation. Overall, the comparable RMSD stability of the NPC1L1-ergosterol complex indicates that ergosterol forms a stable interaction with NPC1L1.

The RMSF analysis demonstrated similar overall flexibility across all three complexes, with distinct regions exhibiting localized fluctuations. In the NPC1L1-ezetimibe complex (black), notable fluctuations were observed at SER332, GLU460, GLY511, SER658, GLU803, and LEU1273. In contrast, the NPC1L1-cholesterol complex (green) exhibited major fluctuations near LEU426, GLY511, PRO974, GLY999, and LEU1273 (Figure 2B). The NPC1L1-ergosterol complex (red) showed prominent peaks at SER332, GLU460, GLY511, ASN975, and LEU1273. All three complexes show comparable flexibility profiles, with only minor fluctuations.

The SASA analysis demonstrated a consistent reduction in solvent exposure for all three complexes, indicating progressive structural compaction throughout the simulation period (Figure 2C). NPC1L1-ezetimibe complex showed SASA in a range of approximately 42,5000 Å^2^ to 39,0000 Å^2^. The NPC1L1-ergosterol ranges in 34,0000-39,5000Å^2^. In contrast, the NPC1L1-cholesterol complex demonstrated a significant reduction in SASA from 45,000 Å^2^ to nearly 41,5000 Å^2^, indicating greater structural tightening. These results indicate that ergosterol and ezetimibe shows similar solvent exposure compared to cholesterol.

Rg analysis was conducted to evaluate the compactness of the ligand-protein complexes, as shown in Figure 2D. The NPC1L1-ezetimibe complex (black) exhibited slight Rg variation, ranging from 35.75 to 36.0 Å, signifying a highly compact and stable conformation during the simulation. In contrast, the NPC1L1-ergosterol complex (red) exhibited comparatively high Rg fluctuations, ranging from 36 to 34.5 Å, indicating increased structural flexibility and dynamic conformational alternations. Similarly, the NPC1L1-cholesterol complex (green) displayed a gradual reduction in Rg from 36.5 Å to 34.5 Å, indicating increased compaction accompanied by structural changes. In comparison to ezetimibe, the NPC1L1-ergosterol complex exhibited more variability in Rg values, signifying enhanced conformational flexibility and reduced structural rigidity.

### 3.5 Molecular mechanics with poisson-boltzmann and surface area solvation (MM-PBSA)

**FIGURE 3:**
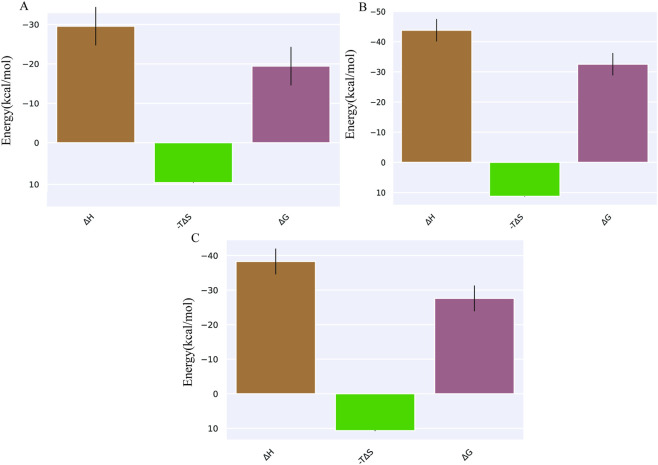
End-state binding free energy calculations of **(A)** NPC1L1-ezetimibe, **(B)** NPC1L1-cholesterol, and **(C)** NPC1L1-ergosterol complexes.

**FIGURE 4:**
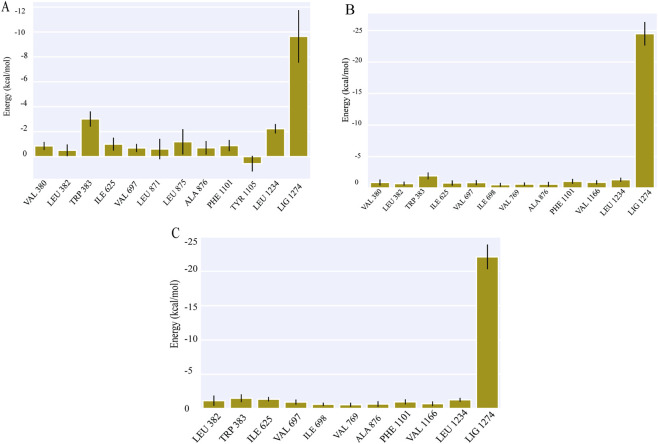
Per-residue binding free energy decomposition analysis of **(A)** NPC1L1-ezetimibe, **(B)** NPC1L1-cholesterol, and **(C)** NPC1L1-ergosterol complexes.

The binding free energy calculations indicated that ezetimibe, cholesterol, and ergosterol form continuous stable interactions with NPC1L1. The binding free energy of the NPC1L1-ezetimibe complex was −19.42 ± 4.87 kcal/mol, while the NPC1L1-cholesterol complex exhibited a more favourable binding free energy of −32.53 ± 3.71 kcal/mol. The NPC1L1-ergosterol complex exhibited a binding free energy of −27.61 ± 3.72 kcal/mol, indicating a binding affinity comparable to that of cholesterol and significantly stronger than that of ezetimibe, as provided in Figure 3; Table 3. Energy decomposition analysis further revealed the contributions of individual amino acid residues to the overall binding energy. The stabilisation of the NPC1L1-ezetimibe complex was primarily localised, with significant energetic contributions from TRP383, ILE625, LEU875, and LEU1234. Similarly, the NPC1L1-cholesterol complex showed strong per-residue contributions from TRP383, ILE625, PHE1101, and LEU1234. In contrast, the NPC1L1-ergosterol complex demonstrated a broader stabilization profile, with significant contributions from TRP383, ILE625, PHE1101, and LEU1234, suggesting a similar per-residue impact on the total binding affinity of all three complexes (Figure 4).

**TABLE 3:** Free energy calculations of A. NPC1L1-ezetimibe, B. NPC1L1-cholesterol, and C. NPC1L1-ergosterol complexes.

Sl.no.	Protein–ligand complex	ΔG bind (kcal/mol)
A	NPC1L1-ezetimibe	−19.42 ± 4.87
B	NPC1L1-cholesterol	−32.53 ± 3.71
C	NPC1L1-ergosterol	−27.61 ± 3.72

## 4 Discussions

NPC1L1 facilitates intestinal absorption of dietary and biliary cholesterol through clathrin-mediated endocytosis (Zeng et al., 2022). Cholesterol binds to its sterol-sensing domain (SSD) located in the extracellular N-terminal domain. This interaction causes NPC1L1, which promotes its interaction with the clathrin/AP2 complex, leading to clathrin-mediated endocytosis. These complexes are then transported to endosomal and lysosomal compartments for cholesterol processing (Davis and Altmann, 2009; Ge et al., 2008). Most of the NPC1L1 is recycled back to the plasma membrane, enabling continuous cholesterol transport (Jia et al., 2011). Inhibition of NPC1L1 significantly reduces intestinal sterol absorption and thereby lowers circulating cholesterol concentrations (Ticho et al., 2021). This specificity highlights the therapeutic value of targeting NPC1L1 in the management of hypercholesterolemia.

NPC1L1 contains four major domains classified as N-terminal domain (NTD), middle luminal domain (MLD), transmembrane domain (TMD), and cysteine-rich domain (CTD), with the sterol-sensing domain (SSD; TM3–TM7) located within the NTD, where it captures extracellular cholesterol for transport toward the membrane. Structural studies detected a continuous tunnel linking the NTD to the SSD, serving as a conduit for sterol translocation into the lipid bilayer. The MLD constitutes the binding site for ezetimibe, which inhibits cholesterol uptake by interrupting this transport pathway. Binding pocket comprises of the neck helices, linker2, linker8, and transmembrane segments (TM5, TM7, TM11, and TM13) (Zhang et al., 2022a). Cholesterol, the natural substrate of NPC1L1, primarily interacts with the N-terminal domain (NTD), which mediates sterol recognition and uptake, while the loop between TM7 and TM8 facilitates cholesterol transport, and the C-terminal tail provides the signal required for endocytosis. However, ezetimibe binds to the loop within the middle luminal domain (MLD) instead and blocks NPC1L1-mediated transport (Zhang et al., 2011; Weinglass et al., 2008).

This study aimed to explore the molecular basis of the NPC1L1-ergosterol interaction and to investigate its potential as a safer alternative treatment for managing hypercholesterolemia and related cardiovascular conditions. An *in silico* evaluation of ergosterol against NPC1L1 was performed, and its binding affinity was compared with ezetimibe and cholesterol. Docking and simulation analyses indicated that ergosterol forms a stable complex with NPC1L1 and remains within the binding site throughout the simulation.

NPC1L1-ezetimibe forms hydrogen bond with LEU875, polar interaction with GLN873 and hydrophobic interactions with VAL380, LEU382, TRP383, ILE525, LEU621, VAL667, ILE698, VAL701, ALA768, VAL769, PHE772, LEU871, LEU875, ALA876, PHE1101, TYR1102, VAL1160, LEU1234, PHE1238, and PHE1239. The NPC1L1-cholesterol complex formed hydrogen bond with TYR1102 hydrophobic interaction with VAL380, LEU382, TRP383, LEU621, ILE625, VAL697, ILE698, VAL701, ALA768, VAL769, PHE772, LEU871, ALA876, PHE1101, VAL1166, ILE1169, LEU1234, and PHE1239, and polar interaction with GLN873.

The NPC1L1-ergosterol complex forms a polar interaction with SER695, hydrogen bond with GLN873 and hydrophobic interaction with VAL380, LEU382, TRP383, LEU621, ILE625, VAL697, ILE698, VAL701, ALA768, VAL769, PHE772, LEU871, ALA876, PHE1101, TYR1102, VAL1166, ILE1169, LEU1234, and PHE1239.

Pharmacokinetic and toxicity analysis using pk-CSM demonstrated that ergosterol adheres to Lipinski’s rule of five and is characterized by high oral bioavailability, favourable distribution, higher intestinal absorption, and minimal toxicity risk. The molecular dynamics simulation results also reinforced the stability and compaction patterns of the three NPC1L1-ligand complexes obtained from docking studies. RMSD and RMSF analysis exhibited moderate deviation and a minor fluctuation of NPC1L1-ergosterol complex in comparison with the NPC1L1-ezetimibe complex. Rg analysis shows more variability in NPC1L1-ergosterol complex, signifying enhanced conformational flexibility compared to NPC1L1-ezetimibe complex. SASA results indicate that NPC1L1-ergosterol and NPC1L1-ezetimibe complex show similar solvent exposure compared to cholesterol.

However, the total ΔG binding free energy for ergosterol was energetically more favourable, with a binding free energy of −27.61 ± 3.72 kcal/mol, and cholesterol was −32.53 ± 3.71 kcal/mol compared to −19.42 ± 4.87 kcal/mol for ezetimibe. Residue-level decomposition revealed contributions from TRP383, ILE625, PHE1101, and LEU1234 in both complexes. Overall, these findings indicate that ergosterol could serve as a promising drug candidate for treating hypercholesterolemia and related cardiovascular conditions.

Site-directed mutagenesis studies have shown that the LEU216 mutation in the N-terminal domain of NPC1L1 disrupts the formation of NPC1L1–flotillin–cholesterol membrane microdomains, thereby inhibiting cholesterol absorption and reducing NPC1L1-mediated uptake in mouse liver and cultured cells (Zhang et al., 2011). Similarly, mutation of TRP347 in transmembrane helix 2 significantly decreases cholesterol uptake efficiency and is associated with reduced cardiovascular disease risk (Long et al., 2021). In addition, mutations at Q873A, F1101A–Y1102A, and F1239R impair ezetimibe binding (Hu et al., 2021). Notably, residue F1101, which is critical for ezetimibe binding as demonstrated by mutagenesis studies, was also found to be involved in ergosterol binding in our computational analysis, suggesting that ergosterol may act as a potential alternative inhibitor with a similar binding mechanism.

Ergosterol possesses an amphipathic structure, characterised by conjugated double bonds and a β-hydroxyl group, which enhances membrane fluidity and stability. Upon ultraviolet exposure, it is converted to vitamin D_2_ (ergocalciferol) and its active form, calcitriol, contributing to calcium and bone regulation (Villares et al., 2012). It exhibits diverse pharmacological properties, including anti-inflammatory activity via modulation of the JAK–STAT pathway and suppression of iNOS and COX-2 (Huan et al., 2017). It also provides neuroprotection by inhibiting TLR4/NF-κB signalling (Kushairi et al., 2020), along with reported anticancer, antidiabetic, antimicrobial, and anti-platelet aggregation properties (El Omari et al., 2024; Hu et al., 2017). Adding to these findings, ergosterol from *Pleurotus ostreatus* shows antidiabetic activity, and its derivatives inhibit *Helicobacter pylori* and *Aspergillus flavus*. Emerging evidence also suggests its role in ameliorating hepatic steatosis, underscoring its therapeutic potential in inflammatory, metabolic, and infectious diseases (Rangsinth et al., 2023). In addition, various wet lab experiments have reported diverse biological functions of ergosterol, especially in lipid regulation. In Sprague–Dawley rats, ergosterol inhibited cholesterol incorporation into micelles, thereby reducing intestinal absorption (He et al., 2019), while experiments in murine models showed substantial reductions in serum cholesterol after ergosterol administration from *Lentinula edodes* (Mo et al., 2019). Extracts of *Pleurotus citrinopileatus* showed antioxidant and antihyperlipidemic properties in hyperlipidaemic hamster models (Hu et al., 2006), whereas clinical investigations on *P. ostreatus* validated its cholesterol-lowering effects in humans. Moreover, ergosterol and its derivatives were reported to have oxygen radical absorbance and cyclooxygenase (COX) inhibition *in vitro* (Schneider et al., 2011), highlighting their therapeutic potential in oxidative stress and dyslipidaemia management.

Building on these findings, mechanistic studies further highlight the role of ergosterol in cholesterol metabolism. In a high-fat and high-sucrose (HFHS) rat model, feeding of ergosterol for 14 weeks significantly reduced plasma LDL-C and total bile acid levels and reduced cholesterol precursor level, while increasing 7-dehydrocholesterol, indicating modulation of the cholesterol biosynthetic pathway (Kuwabara et al., 2023). Similarly, ergosterol has been shown to interfere with the post squalene pathway by altering the expression of key enzymes such as DHCR7 and DHCR24, leading to reduced cholesterol levels and accumulation of sterol precursors (Kuwabara et al., 2022). Furthermore, ergosterol supplementation promotes faecal cholesterol excretion and decreases hepatic cholesterol content, thereby contributing to its overall hypocholesterolaemia effect (He et al., 2019). Collectively, these findings suggest that ergosterol may lower cholesterol levels through competitive inhibition of cholesterol binding to NPC1L1, direct inhibition of NPC1L1, or enhancement of cholesterol excretion; however, the precise mechanism remains unclear.

Adding to this, various studies have reported that NPC1L1 also facilitates the uptake of several fat-soluble compounds, such as vitamin E, vitamin K, and other lipophilic molecules such as coenzyme Q10 (Nashimoto et al., 2020; Takada et al., 2015; Yamanashi et al., 2017; Aizawa, 2025). Accordingly, excessive ergosterol intake may affect the absorption of these compounds, highlighting the importance of further studies and careful dose standardization.

Despite these functional insights, the available computational literature on NPC1L1 remains limited and primarily focuses on non-sterol scaffolds, with no sterol-centric comparisons relevant to ergosterol. For instance, salvianolic acid C from traditional Chinese herbs (Huo et al., 2014) and indole diterpenoids from *Aspergillus* sp. (Wang et al., 2023) have been proposed as a potential inhibitor of NPC1L1. However, these studies do not evaluate phytosterols or examine sterol-specific binding features within the NPC1L1 recognition pocket.

In addition to these findings, other natural and synthetic substances have been investigated as inhibitors of NPC1L1. *In vitro* studies have identified several phytochemicals, such as cinaciguat (Jia et al., 2023) isoliquiritigenin from *Glycyrrhiza glabra* (Zeng et al., 2022), parthenolide from *Tanacetum parthenium* (Liu et al., 2022), and bioactive compounds from coffee leaf extracts (Sansri et al., 2024), as potential NPC1L1 inhibitors, highlighting the increasing interest in both natural and synthetic molecules that target this transporter. Findings from an *in silico* study by Hernández et al. (Zhang et al., 2022a) showed that peptides derived from black soybean and cowpea exhibited stronger binding affinity for NPC1L1 than ezetimibe, suggesting promising leads for future therapeutic development.

The present study is limited by its reliance on computational approaches, which may not fully capture the complexity of biological systems. Additionally, *in silico* analyses do not allow definitive differentiation between a competitive inhibitor and a substrate. Experimental validation via *in vitro* and *in vivo* research is crucial for to determine whether the expected interactions result in physiologically relevant cholesterol-lowering benefits.

## 5 Conclusion

Our study presents comprehensive *in silico* findings demonstrating that ergosterol may serve as a safer alternative for managing hypercholesterolemia and related cardiovascular conditions. Molecular docking, molecular dynamics simulations, and post-MD analysis indicated that the binding affinity and structural stability of ergosterol are comparable to FDA-approved drug, ezetimibe. Furthermore, its pharmacokinetic properties revealed favourable ADMET characteristics, strengthening its potential as a viable drug candidate. These findings are consistent with animal studies indicating that consumption of ergosterol reduces cholesterol levels. Nevertheless, *in vitro* and *in vivo* investigations are required to validate these computational predictions and to establish their clinical relevance in cholesterol regulation.

## Data Availability

The original contributions presented in the study are included in the article/Supplementary Material, further inquiries can be directed to the corresponding author.
