# Supplementary material for: Ergosterol as a natural modulator of intestinal cholesterol absorption via NPC1L1: an in silico insight into hypercholesterolemia
Source: Front Bioinform. 2026 Apr 10;6:1734995. doi: 10.3389/fbinf.2026.1734995 (PMC13106451; doi:10.3389/fbinf.2026.1734995)
Supplement: Supplementary file 1 [file Supplementaryfile1.docx]

**Validation of minimization and equilibration of MD Simulation**

Figure 1A depicts the total potential energy as a function of simulation time for all systems. An initial sharp decrease in energy indicates effective energy minimization and early equilibration, followed by convergence to minor fluctuations. The absence of abrupt changes during equilibration and production phases confirms that all systems achieved a stable energetic state. The overlapping energy profiles further indicate consistent equilibration across simulations.

Temperature and pressure equilibration plots have also been included to support the overall stability of the simulations. During NVT equilibration, the temperature of all systems rapidly converged to the target 300 K and remained stable, with only minor random fluctuations (±3–5 K) indicating effective thermal control. Similarly, during NPT equilibration, temperatures were consistently maintained around 300 K. The comparable temperature profiles across all systems confirm consistent thermal equilibrium prior to production MD simulations (Figure 1B).

During NPT equilibration, the pressure showed large instantaneous fluctuations, as expected for atomistic MD simulations, but remained centered around the target value of 1 bar with no systematic drift, indicating proper barostat performance. After the initial equilibration phase, pressure fluctuations stabilized, and similar pressure distributions across all systems confirmed consistent and reliable pressure coupling. Together with stable energy and temperature profiles, these results demonstrate successful pressure equilibration prior to production MD simulations (Figure 1C).

**Supplementary Figure 1.** Energy minimization and equilibration of the simulated systems. (A) Total potential energy, (B) temperature, and (C) pressure profiles demonstrating successful minimization and equilibration prior to production MD simulations.


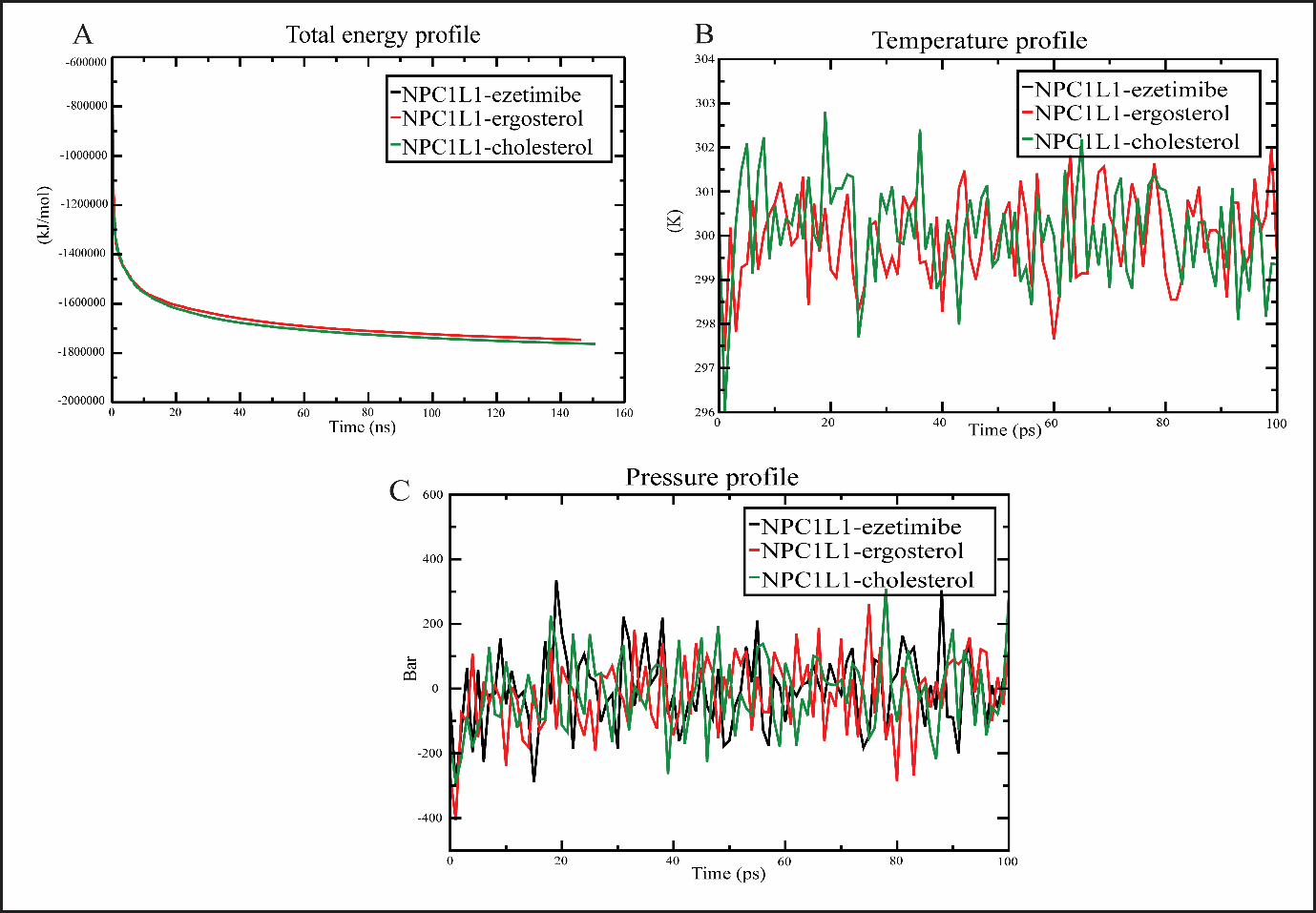


**Running Average (100 Frames) Analysis**


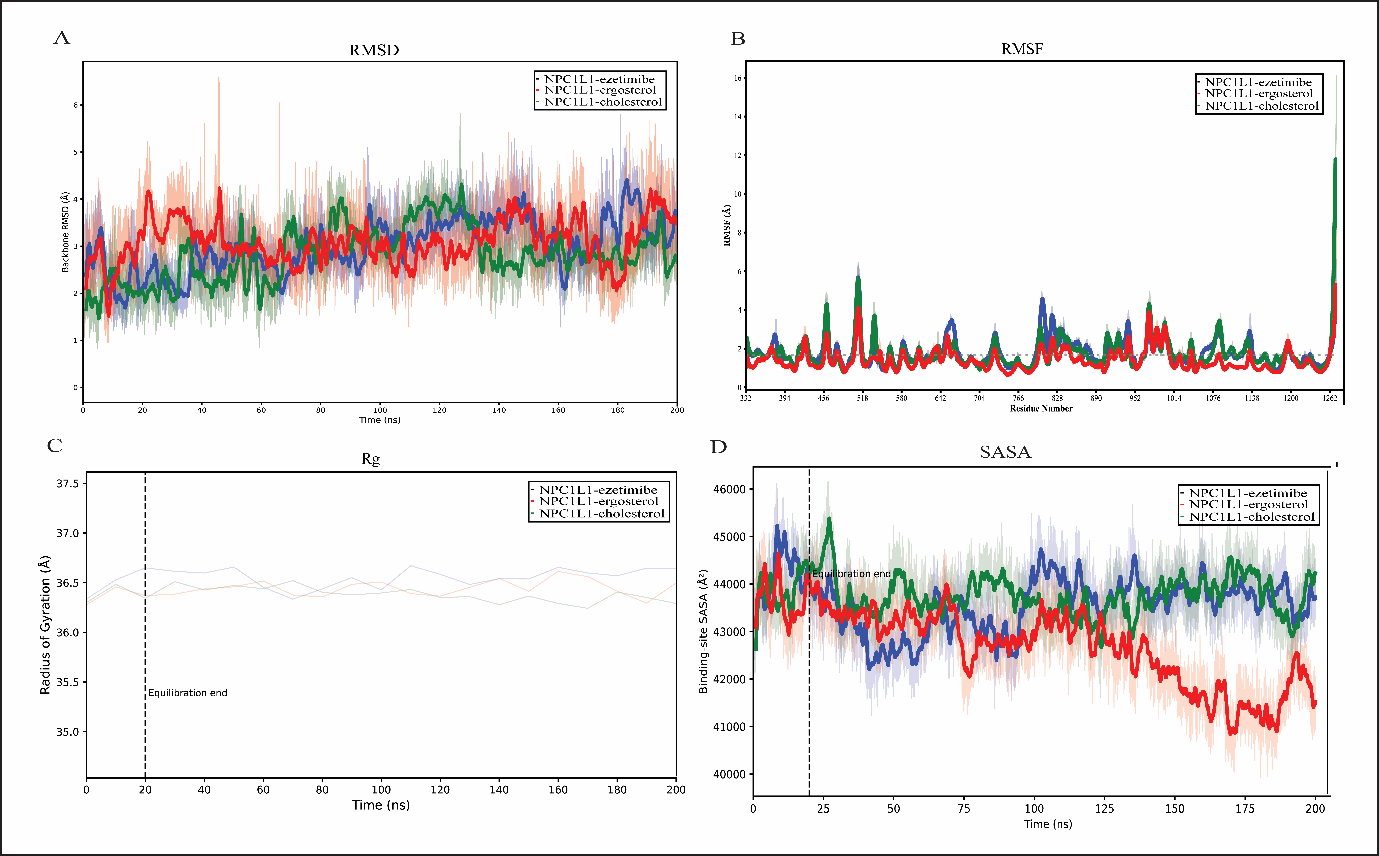


**Supplementary Figure 2.** Running average of 100 frames for (A) RMSD, (B) RMSF, (C) Rg, and (D) SASA

Backbone RMSD analysis was performed to evaluate the structural stability of the protein in complex with EZE, ergosterol, and CLR over a 200-ns simulation (Figure 2B). All systems show an initial rise in RMSD during the early phase, followed by stabilization, indicating successful equilibration. Among the three complexes, the EZE-bound system exhibits the lowest average RMSD and reduced fluctuations throughout the production phase, suggesting enhanced backbone stability. The ergosterol-bound complex shows intermediate stability, while the CLR-bound system displays higher RMSD values and broader fluctuations, indicating increased conformational flexibility. These observations suggest that EZE binding stabilizes the global protein structure more effectively than ergosterol and CLR, which may contribute to improved binding persistence and functional integrity during the simulation timescale.

Residue-wise Cα RMSF analysis shows that the EZE-bound complex exhibits reduced flexibility across several regions compared to the ergosterol- and CLR-bound systems, indicating enhanced local stabilization. Higher RMSF values observed in the CLR-bound complex reflect increased loop flexibility. Large fluctuations at the N- and C-termini are expected due to solvent exposure and do not influence the structured core of the protein. Reduced RMSF in binding-site–adjacent regions of the EZE complex suggests ligand-induced stabilization, consistent with the RMSD results (Figure 2B).

The radius of gyration remains stable for all complexes, indicating preservation of the global fold throughout the simulation. The EZE-bound complex exhibits slightly lower mean Rg values with reduced fluctuations compared to ergosterol and CLR, suggesting tighter packing and reduced global breathing motions. In contrast, the CLR-bound system shows marginally higher Rg values and increased fluctuations, indicative of enhanced conformational flexibility (Figure 2C).

Binding-site SASA analysis was performed to evaluate ligand-induced modulation of pocket solvent exposure (Figure X). The CLR-bound complex exhibits a pronounced and progressive reduction in SASA during the production phase, indicating substantial burial of the binding pocket. In contrast, the ergosterol-bound system maintains higher SASA values, reflecting a more solvent-exposed and flexible pocket conformation.

The EZE-bound complex shows intermediate SASA values with reduced fluctuations, suggesting a stable yet partially accessible binding pocket. These trends are consistent with RMSF and Rg analyses and indicate ligand-dependent regulation of binding-site dynamics that may influence binding stability and functional behavior (Figure 2D).
